# 3D Comparative Evaluation of Condylar Morphology Between Chronic Areca Nut Chewers and Nonchewers: Protocol for a Case-Control Study

**DOI:** 10.2196/84038

**Published:** 2026-03-06

**Authors:** Vaishnavi Tarpe, Suwarna Dangore Khasbage

**Affiliations:** 1 Department of Oral Medicine and Radiology Sharad Pawar Dental College and Hospital Datta Meghe Institute of Higher Education and Research Wardha India

**Keywords:** areca nut, cone beam computed tomography, CBCT, condyle, condylar morphology, temporomandibular joint

## Abstract

**Background:**

Areca nut (AN) is a commonly consumed psychoactive substance, especially in South and Southeast Asia. Chronic chewing of AN has been linked to multiple health problems, including temporomandibular joint (TMJ) disorders. Excessive strain on TMJ during chronic AN chewing can lead to repetitive injury, resulting in microtrauma and macrotrauma to both the TMJ and the surrounding masticatory structures. Previous studies have reported the long-term impact of AN chewing on TMJ by using conventional 2D imaging.

**Objective:**

This study aims to evaluate and compare condylar morphology in chronic AN chewers and nonchewers by using 3D imaging.

**Methods:**

This study will include 90 patients who will be divided into 2 groups: chronic AN chewers (n=45, 50%) and nonchewers (n=45, 50%). The study will be undertaken after obtaining institutional ethics committee approval and written informed consent from each patient. A detailed habit history of all the participants will be recorded. Each patient will undergo a clinical examination and radiographic evaluation of condylar morphology. Condylar morphology will be evaluated using cone beam computed tomography scans in both sagittal and coronal planes. All the findings will be recorded and then examined for statistical significance.

**Results:**

On comparison of condylar morphology between chronic AN chewers and nonchewers by using cone beam computed tomography, statistical variations relevant to structural and pathological alterations such as osteophytes, surface flattening, and erosions are likely to occur.

**Conclusions:**

This study aims to overcome the limitations of conventional 2D radiography and provide a more accurate assessment of condylar morphology. The findings should fill an existing gap in the literature by providing useful insights on the effects of chronic AN chewing on condylar structure by using 3D imaging. This research may help to improve the diagnosis, prevention, and management of TMJ disorders.

**Trial Registration:**

Clinical Trials Registry-India CTRI/2025/06/088238; https://ctri.nic.in/Clinicaltrials/pmaindet2.php?EncHid=MTMzNzQz&Enc=&userName=

**International Registered Report Identifier (IRRID):**

PRR1-10.2196/84038

## Introduction

Areca nut (AN) has a long history of use. A number of factors contribute to the popularity of AN chewing addiction, such as social acceptance, religious convictions, and perceived health advantages. AN is an ingredient in a variety of items, including *khaini*, *pan masala*, *mawa*, *paan*, and *gutkha*. AN (generally referred to as *Areca catechu* L and betel nut) is the fourth most frequently used psychoactive drug all over the world, after alcohol, nicotine, and caffeine [1].

AN is chewed for various reasons, such as pain treatment, euphoria, refreshment, and increased salivation. Although AN chewing is a highly common habit in Southeast Asia, its use has expanded to North America and Europe. The fruit of the *Catechu* tree produces AN as an endosperm. It is composed of tannins and alkaloids, which include arecoline, arecolidine, guvacine, guvacoline, and arecaidine. Regular consumption of AN may be harmful to one’s health. The risk of oral cancer increases with the frequency and duration of AN chewing, highlighting AN as a significant carcinogen. As a psychoactive substance, chronic AN use is categorized by the World Health Organization as a Group 1 human carcinogen, as regular AN chewing is associated with an increased risk of oral squamous cell carcinoma estimated at 2.3%-7.6%. The associated mortality risk is substantial, making this behavior clinically noteworthy [2,3].

Its principal alkaloid, arecoline, is responsible for systemic effects, including cardiovascular disturbances, metabolic disorders such as type II diabetes, immune dysregulation, neurotoxicity, respiratory impairment, and reproductive complications. An association between AN chewing and an increased risk of liver disease has been reported, with several studies demonstrating an additive effect on individuals who are positive for hepatitis B or C. Given its extensive adverse health consequences, AN use poses a significant public health concern [4,5].

Chronic chewing of AN, often kept in the mouth for hours, has been associated with various oral health problems. These include lesions of the mucosa, staining of teeth, dry mouth, periodontal diseases, and plasma cell stomatitis. Furthermore, it contributes to masticatory muscle pain and temporomandibular joint disorders (TMDs), which are common conditions affecting the craniofacial area. The overuse of the temporomandibular joint (TMJ) due to prolonged AN chewing is likely to cause microtrauma and macrotrauma to the TMJ and the masticatory apparatus [6].

Repetitive and prolonged chewing habits, such as AN, betel quid, and tobacco use, subject the masticatory muscles to sustained excessive forces, leading to masseter muscle hypertrophy, a benign condition characterized by an increase in the size of the masseter muscle, which may be unilateral or bilateral. With continued exposure, this mechanical overload results in increased muscle prominence and altered echogenicity of the masseter muscle on imaging [7,8].

Although there is some debate over its relationship with TMD, tooth wear is linked to cumulative parafunctional and oral functions. While modern studies show no clear relationship between tooth loss and TMJ changes, a correlation has been observed in Aboriginal and prehistoric populations. Some studies have claimed that parafunctional habits, including bruxism, clenching, and chewing gum or qat, may be linked to TMD [9].

One study examined the association between occlusal tooth wear and TMD by using the Smith and Knight Tooth Wear Index. Tooth wear was more common in patients with TMD, particularly grade 1 wear in the 26- to 40-year age group and in females, with posterior teeth most frequently affected. TMD is common in young adults and is significantly associated with functional and static occlusal parameters. These findings highlight the importance of evaluating occlusal factors for early diagnosis of TMD. On the basis of the TMD diagnostic criteria, previous studies have hypothesized that excessive tooth wear caused by chronic AN chewing is linked to TMD [9-11].

Mandibular condyle is a crucial bony structure that is often affected by TMD. It forms an articulation with the glenoid fossa, facilitating the movement of the mandible. The mandibular condyle is also the primary growth site in the development of the mandible. Normally, the condyle has a broadly ovoid form and measures approximately 8 mm to 10 mm anteroposteriorly and 15 mm to 20 mm mediolaterally. However, its morphology significantly fluctuates among different individuals and across different age groups. The shape and morphology of the condyle can change due to numerous factors such as normal developmental variability, developmental diseases, tooth wear, parafunctional habits, malocclusion, condylar remodeling, trauma, and disorders of the TMJ [12]. Tooth wear, regardless of its cause, disrupts the normal balance of force distribution, leading to structural adaptation within the condyle. These changes might not show any clear clinical symptoms [9].

A complete evaluation of structural changes in the condyle requires 2 steps: clinical examination and radiographic assessment to confirm the diagnostic findings. Orthopantomograms and transcranial views are some conventional radiographic techniques that have been used to examine the TMJ. However, these 2D images have limitations such as the superimposition of surrounding structures. In contrast, 3D imaging modalities, such as computed tomography (CT), magnetic resonance imaging (MRI), and cone beam CT (CBCT), offer a clearer view of condylar morphology, allowing a more accurate assessment of the TMJ [13].

CT imaging provides high-resolution, detailed images of bony structures. It effectively identifies bone abnormalities such as fractures, osteoarthritis, or degenerative changes in the TMJ. However, CT involves a high-exposure dose of ionizing radiation. It also has drawbacks in showing soft tissues, such as the articular disc, muscles, and ligaments, which are important for diagnosing TMD. Furthermore, CT can be expensive and may be impacted by artifacts from dental hardware, reducing image clarity [14].

MRI is a valuable tool for visualizing the TMJ, as it can produce clear images of soft connective tissues. However, MRI is not the best choice for checking bony abnormalities or fractures in the TMJ, as it is less effective for imaging bones compared to CBCT. Additionally, patients with metal implants, such as certain dental appliances or prostheses, may not be able to undergo an MRI due to dangers associated with the magnetic field [15].

CBCT is a reliable and effective imaging method for viewing craniofacial structures. This 3D imaging technique provides detailed structural information without overlapping or distortion. It is also preferred because it uses a relatively low radiation dose compared to conventional CT, making it safer for patients. Moreover, CBCT scans are more affordable than conventional CT scanners, making them a cost-effective choice for many dental and orthopedic practices. This combination of detailed imaging, safety, and efficiency makes CBCT an excellent tool for evaluating the shape of the condylar head [16,17].

Numerous studies have investigated the effect of chronic AN chewing and showed significant effects on oral tissues, including the oral mucosa and hard tissues [2]. However, a literature search revealed that few studies have focused on changes in condylar morphology among chronic AN chewers. Therefore, this study aims to use CBCT to compare the condylar morphological changes between chronic AN chewers and nonchewers, addressing the paucity of research on the condylar morphological changes associated with chronic AN chewers.

## Methods

A case-control study comprising 90 patients (chronic AN chewer: n=45, 50%) and nonchewer: n=45, 50%) will be conducted between September 2025 and May 2027.

### Ethical Considerations

The study will be carried out in Oral Medicine and Radiology department at Sharad Pawar Dental College and Hospital, after receiving approval from the "Institutional Ethics Committee (IEC)" of Datta Meghe Institute of Higher Education and Research (Deemed to be University), Sawangi (Meghe), Wardha, India [Approval No. DMIHER (DU)/IEC/2025/694)] and will be conducted in accordance with the Declaration of Helsinki. The patients, who are willing to participate and provide written informed consent will be included for the study. Participation is voluntary, and all participants may withdraw from the study at any time without any effect on their clinical care. All collected data will be fully de-identified prior to analysis and stored on password-protected, access-restricted institutional systems to ensure confidentiality and data security. No financial compensation will be provided to study participants. CBCT imaging in prospectively enrolled AN chewers will be performed only when clinically indicated and not solely for research purposes, in accordance with the ALARA (As Low As Reasonably Achievable) principle. No additional radiation exposure will be incurred for the control group, as their CBCT scans will be retrospectively analysed. All exposure parameters will be standardized and documented [18-20].

### Eligibility Criteria

#### Inclusion Criteria

Patients aged 18 to 40 years with a habit of chronic AN chewing for more than 1 year and a complete set of permanent dentition, excluding third molars, will be included in the study. Patients showing clinical evidence of generalized attrition and having a normal skeletal relationship will be considered for inclusion. Patients in the control group will have all permanent teeth except wisdom teeth, no history of AN chewing, and no evidence of attrition or reduced vertical height. Tooth wear will be assessed as a covariate by using the Smith and Knight Tooth Wear Index and will not be considered a criterion for group allocation [21].

#### Exclusion Criteria

Individuals who have undergone TMJ surgery or experienced a fracture, those with congenital TMJ anomalies, patients with a history of trauma from occlusion, those with systemic diseases affecting bone health, patients taking medications that impact bone health, and those with pathological lesions in the jaw or developmental tooth anomalies will be excluded.

### Study Procedure

The first step will be a detailed assessment of habit history. This will include chewing frequency per day, average duration of each chewing episode, total years of use, chewing-side preference, and type of AN product consumed. A cumulative “chewing-load index” will be calculated by combining frequency, daily duration, and total years of chewing to create a standardized measure of exposure. Additionally, an analysis of a subgroup within the AN chewer group will compare condylar morphology between the predominant chewing side and the nonchewing side. This will help to evaluate the mechanical effects related to each side. This method improves the measurement of exposure and boosts the study’s analytical power.

A complete medical history will also be gathered to rule out any systemic diseases affecting joints, medications influencing joint structure, and any history of trauma or surgery that impacts the TMJ.

A comprehensive clinical examination will be performed for each patient. The TMJ will be examined to identify any disorders. The assessment will include the following:

- Joint movement inspection—evaluation of joint movement was done during mouth opening and closing to detect restricted mouth opening, pain, deviation, or deflection.
- Palpation—palpation of the joint will be performed to assess clicking sounds, crepitus, and tenderness during jaw movements.

Thorough intraoral examination will be done for patients with decreased vertical height due to AN chewing and loss of tooth structure. Tooth structure loss will be recorded based on the Smith and Knight Tooth Wear Index [22].

Each chronic AN chewer will then be evaluated for condylar morphology by performing CBCT. Imaging will be performed using a Planmeca ProMax 3D unit (Planmeca Oy; equipment ID: G-XR-109125), with the following parameters: 90 kV, 6.3 mA current, exposure time of 12.056 seconds, field of view of 5×5 cm, dose-area product of 447 mGy·cm², 251 slices, voxel size of 200 µm, rotation angle of 360°, pixel size of 200 µm, and software version 3.9.6.152. All AN nonchewers will be evaluated retrospectively, and their dental and medical history, clinical records, and radiographic data (CBCT) will be available.

**Figure 1:**
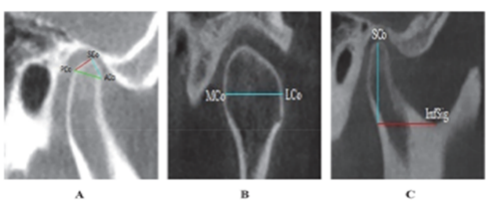
Cone beam computed tomography images images showing condylar morphology measurements: (A) condylar length between ACo–PCo, (B) condylar width between MCo–LCo, and (C) condylar height from SCo to the sigmoid notch. ACo: anterior condyle point; PCo: posterior condyle point; MCo: medial condyle poles; LCo: lateral condyle poles: SCo: superior condyle point.

Condylar morphology will be evaluated using CBCT scans in both sagittal and coronal planes. To calculate the length of the condyle, a line will be drawn connecting the anterior mandibular condyle point and the posterior mandibular condyle point. These points will be situated 4 mm below the superior mandibular condyle point on either side of the condyle [6]. Width of the condylar will be measured in the coronal plane as the linear distance between the medial and lateral mandible poles [6]. In contrast, height of the condyle will be determined as a perpendicular linear distance from the superior mandible condyle to a line drawn between the most inferior point of the sigmoid notch and the tangent of the posterior surface of the ramus in the sagittal plane [6], as shown in Figure 1.

### Bias Mitigation

To reduce possible sources of bias, several steps will be taken during the study. Controls will be matched to cases based on age and sex to limit confounding. All participants will receive CBCT imaging with the same machine settings, whenever possible. Retrospective scans will be included only if they were obtained using the same equipment and parameters and fulfill the inclusion criteria. All scan details, such as machine type, 90 kilovoltage (kV), tube 6.3 current (mA), voxel size, field of view, and scan date, will be recorded. Image evaluation will be done by 2 radiologists who are blinded to the participants’ habit history. They will use standardized scoring sheets and repeated measurements to ensure consistency between raters. In addition to matching, multivariable analyses will adjust for factors that may differ between groups, such as age, sex, degree of attrition, and duration of the chewing habit. Sensitivity analyses will also be done to check if the findings remain consistent when we focus on scan acquisition parameters. Finally, we will clearly describe the study design, potential biases, and limitations linked to mixed data sources to guarantee transparent reporting.

### Sample Size

The sample size for this investigation was determined based on the expected difference in continuous condylar morphometric measurements between groups. The entire sample size estimation procedure, including the full statistical formula and parameter assumptions, is described in Multimedia Appendix 1 [6,23]. On the basis of these predictions, at least 45 participants will be needed per group (chronic AN chewers and nonchewers).

### Statistical Methods

This study will compare chronic AN chewers and nonchewers by using random sample selection and strong statistical methods, including the Student 1-tailed *t* test, 1-way ANOVA, and chi-square test. SPSS (version 27.0; IBM Corp) and GraphPad Prism (version 7.0; GraphPad Software) will be used for data analysis, and the Nawaz et al formula will be applied to determine sample size [24]. To ensure scientific reliability, the analytical strategy was adapted from the study by Almashraqi et al [6].

### Reliability Assessment

A reliability plan has been included to ensure measurement accuracy. Two radiologists who had attended calibration sessions standardized landmark identification and measurement techniques. This will be according to the principles used in the study by Almashraqi et al [6]. They will assess all CBCT scans independently by using a standardized protocol. Intraobserver and interobserver reliability will be evaluated on 20% of randomly selected scans by using intraclass correlation coefficients, with values ≥0.80 considered acceptable. Any discrepancies will be reviewed and resolved by consensus to ensure consistent and reproducible measurements.

### Data Collection, Management, and Analysis

#### Plan of Data Collection

Patients who report to the Department of Oral Medicine and Radiology will have their AN chewing habit assessed. Following diagnosis, the patients will provide written informed consent and receive a comprehensive explanation of the intervention process. After consent has been obtained, CBCT will be used to analyze the AN chewer prospectively. This will create the following 2 distinct groups: AN chewers (45/90, 50%) and nonchewers (45/90, 50%), based on the patient’s habit history and comprehensive clinical examination. To improve transparency and reproducibility, the manuscript includes clearer and more organized reporting of the study design, participant recruitment, and flow. It also defines outcome measures. Additional details specify how CBCT imaging will be done, how clinical and habit-related data will be collected, and how all measurements will be recorded and analyzed. The procedures for radiologists, who were blinded to chewing status and attended calibration sessions, reliability testing, and statistical analysis, have also been described in more detail. This enhances methodological clarity and ensures that the study protocol is fully reproducible. All the results will be documented in a standardized tabular manner, as shown in Multimedia Appendix 2.

#### Data Collection and Retention

Proper counseling regarding the disease will increase the patient’s participation in the study.

#### Data Management

A master data sheet will be created to include the CBCT image data of the AN chewers and nonchewers, along with the difference in condylar morphology and pathological alterations in each group. All collected data will be assigned coded identifiers, and no personal information will be stored with the clinical or CBCT records. Digital files, including scan data and measurement sheets, will be stored on password-protected institutional servers. Access will be limited to authorized study investigators only. Data will be backed up regularly on secure, encrypted systems to prevent loss or unauthorized access. These measures ensure confidentiality, data integrity, and adherence to institutional ethical standards.

### Data Monitoring

Oversight of data accuracy, protocol compliance, and ethical adherence will be maintained by the Department of Oral Medicine and Radiology under the supervision of the guide and the IEC. No interim analyses or stopping guidelines are applicable to this observational study. Data will be analyzed only after completion of enrollment and imaging.

## Results

Only individuals fulfilling the inclusion and exclusion criteria will be considered as study subjects. This study is a registered, unfunded protocol study (CTRI/2025/06/088238) and is currently in the pre-recruitment phase. Participant enrollment and data collection are scheduled to be conducted from September 2025 to May 2027. As of the time of manuscript submission, no participants have been recruited and data analysis has not yet commenced. The primary outcome will evaluate condylar morphological changes associated with chronic areca nut (AN) chewing, while secondary outcomes will assess pathological alterations of the condyle, including osteophyte formation with subcortical sclerosis, articular surface flattening of the superior condylar surface with subcortical sclerosis, abnormal condylar shape, and surface erosion. The results are expected to provide insights into the functional impact of chronic AN chewing on the temporomandibular joint and may aid in early identification of pathological condylar changes. Study results are anticipated to be published after completion of data analysis in 2027.

## Discussion

Chronic AN chewing has been extensively associated with oral mucosal lesions. However, its impact on the TMJ, particularly on condylar morphology, remains insufficiently studied using CBCT. Almashraqi et al [6] investigated CBCT findings in the TMJ of individuals with a history of chronic qat chewing (QC). They examined TMJ dimensions and osteoarthritic alterations on CBCT. According to the qualitative CBCT results, QC had substantially more osteoarthritic alterations than non qat chewers, including osteophyte, subcortical sclerosis, articular surface flatness, and subcortical cysts. Quantitative assessments of condylar dimensions showed no significant difference among both categories. Notably, the chewing side of individuals with QC displayed more alterations than the nonchewing side. The findings show that chewing qat has negative effects on the TMJ, primarily in the form of osteoarthritic alterations [6].

Liu et al [9] performed a study to investigate the relation between severe tooth wear caused by betel nut chewing and the development of TMD. Age, sex, and severely worn dentition due to betel nut chewing were significant factors contributing to the overall occurrence of TMD. Multivariable analysis revealed a significant, dose-dependent association between intra-articular TMD and badly worn dentition resulting from betel nut chewing, indicating a direct relationship between the severity of tooth wear and the likelihood of developing intra-articular TMD [9].

Al-koshab et al [22] investigated the morphology of the condyle and glenoid fossa by using CBCT in South-East Asians. Condylar length and roof of the glenoid fossa (RGF) thickness did not differ significantly by sex; however, male participants had significantly larger condylar volume, width, height, and joint gaps. While the mean left condylar height and RGF thickness were higher, the mean right TMJ condylar volume, width, and length were significantly higher when comparing the 2 TMJs. With the exception of condylar height, which is larger in Chinese participants, we could not find any significant differences between the 2 ethnic groups’ condylar measures and RGF thickness [22].

Khalid Nawaz et al [24] conducted a clinical study on TMJ dysfunction associated with betel nut chewing and reported that long-term chewing of betel nuts can lead in TMJ dysfunction syndrome, as the masticatory forces are transferred to the TMJ. Chewing betel nuts for an extended period can result in TMJ dysfunction syndrome. The consistency of the betel nut is another important factor in TMJ dysfunction syndrome. Clicking and preauricular pain appeared to be the most common symptoms across all age groups. They concluded that the incidence in female participants was greater than that in male participants [23].

Larheim et al [25] conducted a study on TMJ diagnostics by using CBCT. This study provides an updated perspective on the role of CBCT in evaluating TMJ conditions. The study highlights the diagnostic accuracy and value of CBCT compared to other imaging methods. CBCT is a cost-effective and dose-efficient tool for assessing different TMJ issues, such as osteoarthritis, juvenile osteoarthritis, and rheumatoid arthritis. CBCT offers better images of bone structures than conventional radiographs and MRI scans [25].

## Appendix

Multimedia Appendix 1 Sample size calculation.

Multimedia Appendix 2 Data on the morphological and pathological changes of the condyle.

Multimedia Appendix 3 SPIRIT 2025 checklist.

## Abbreviations

AN: areca nut
CBCT: cone beam computed tomography
CT: computed tomography
IEC: institutional ethics committee
MRI: magnetic resonance imaging
RGF: roof of the glenoid fossa
TMD: temporomandibular disorder
TMJ: temporomandibular joint
QC: qat chewing

## Data Availability

Deidentified datasets created during this study will be deposited in a publicly accessible research data repository after completion of data collection and analysis. Before public release, the data will be available from the corresponding author on reasonable request for research purposes, subject to institutional approval and data-sharing agreements. Any additional materials, including coding sheets or measurement templates, will be provided as multimedia appendices. No identifiable participant information will be shared.
