# Supplementary material for: 3D Comparative Evaluation of Condylar Morphology Between Chronic Areca Nut Chewers and Nonchewers: Protocol for a Case-Control Study
Source: JMIR Res Protoc. 2026 Mar 6;15:e84038. doi: 10.2196/84038 (PMC13005062; doi:10.2196/84038)
Supplement: Multimedia Appendix 1 [file resprot_v15i1e84038_app1.docx]

**Multimedia Appendix 1: Sample Size Calculation**

The sample-size estimation was conducted using continuous outcome measures derived from CBCT-based condylar morphometry.

**Sample size formula for difference between two groups:**

The sample size is calculated as:

n = (Z_α/2_+Z_β_)^2^ * (p_1_(1-p_1_)+p_2_(1-p_2_)) / (p_1_-p_2_)^2^,

where

Z_α/2_ is the critical value of the Normal distribution at α/2 (e.g. for a confidence level of 95%, α is 0.05 and the critical value is 1.96) =1.96

Z_β_ is the critical value of the Normal distribution at β (e.g. for a power of 80%, β is 0.2 and the critical value is 0.84) =0.84

p_1_ and p_2_ are the expected sample proportions of the two groups.

P1 = Proportion of Osteotype in AN chewer

=51.2% =0.512

P2= Proportion of Osteotype in non-AN chewer

C

= 22.7% =0.227

n = (1.96+0.81)^2^ * (0.512(1-0.512)+0.227(1-0.227)) / (0.512-0.227)^2^

= 41.05 = 45 patients needed in each group

**Study Reference :** Abeer A. Almashraqi et al

***Formula Reference :*** *Wang, H. and Chow, S.-C. 2007. Sample Size Calculation for Comparing Proportions. Wiley Encyclopedia of Clinical Trials.*

**Statistical Formulas : S**tudent’s t-test, one way ANOVA, Chisquare test

**Software used :** SPSS 27.0 version and GraphPad Prism 7.0 version

**Study Design :** Comparative study design(Chewers and Non Chewers)

**Sample Allocation:** Random selection of samples
