# Supplementary material for: 3D Comparative Evaluation of Condylar Morphology Between Chronic Areca Nut Chewers and Nonchewers: Protocol for a Case-Control Study
Source: JMIR Res Protoc. 2026 Mar 6;15:e84038. doi: 10.2196/84038 (PMC13005062; doi:10.2196/84038)
Supplement: Multimedia Appendix 2 [file resprot_v15i1e84038_app2.docx]

**Table S1: CBCT findings on Sagittal and Coronal sections**

| **Sections** | **Sagittal** | | **Coronal** | |
| --- | --- | --- | --- | --- |
| **Measurements of condyle** | **(mm)** | | **(mm)** | |
| 1. Condylar Length |  | |  | |
| 1. Condylar Width |  | |  | |
| 1. Condylar Height |  | |  | |
| **Pathological alterations:** | **Present** | **Absent** | **Present** | **Absent** |
| Osteophyte with subcortical sclerosis |  |  |  |  |
| Articular surface flattening in the superior surface of the condyle with subcortical sclerosis |  |  |  |  |
| Articular surface flattening with abnormal condylar shape |  |  |  |  |
| Surface erosion |  |  |  |  |
